# The Impact of Classical Cardiovascular Risk Factors on Hospitalization and Mortality among Hajj Pilgrims

**DOI:** 10.1155/2023/9037159

**Published:** 2023-04-18

**Authors:** Meity Ardiana, Eka Rahayu Utami, Makhyan Jibril Al Farabi, Yusuf Azmi

**Affiliations:** Department of Cardiology and Vascular Medicine, Faculty of Medicine, Universitas Airlangga-Dr Soetomo General Hospital, Surabaya 60132, Indonesia

## Abstract

**Background:**

Cardiovascular disease (CVD) is the leading cause of morbidity and mortality during Hajj. The objective of the present study was to examine the effect of classical cardiovascular disease risk factors on mortality and hospitalization among Hajj pilgrims from East Java, Indonesia, during 2017, 2018, and 2019.

**Methods:**

This study was a retrospective cohort of Hajj pilgrims from East Java, Indonesia, from 2017 to 2019. The data on risk factors were obtained from the pre-embarkation Hajj screening records. The diagnosis of hospitalization and cause of death during the Hajj period were obtained from the medical report and hospital/flight doctor death certificate.

**Results:**

A total of 72078 eligible subjects were included in this study. 33807 (46.9%) were men, and 38271 (53.1%) were women, and the majority (35%) were aged between 50 and 59 years. A total of 42446 pilgrims (58.9%) were classified as high risk due to underlying health conditions such as hypertension, diabetes, or if they were aged 60 years or older. The overall hospitalization rate is 971 per 100,000 pilgrims and the overall death rate is 240 deaths per 100,000 pilgrims. Multivariate analysis using logistic regression showed that male gender, age > 50 years, hypertension grade II-III, diabetes, overweight, and obesity were associated with a higher risk of hospitalization. Moreover, male gender, diabetes, and overweight were associated with a higher risk of mortality. Of all hospitalized patients, 92 patients (13.1%) had an initial diagnosis of CVD, and CVD is the main cause of mortality (38.2%) of pilgrims.

**Conclusion:**

Pilgrims with classical cardiovascular risk factors were associated with increased hospitalization and mortality.

## 1. Introduction

Hajj, an annual pilgrimage in the Kingdom of Saudi Arabia (KSA), is the largest annual global temporary migration and largest mass gathering event in the world, performed by more than 2 million Muslims from more than 183 countries [1]. Indonesia, as the country with the largest Muslim population, holds the largest Hajj visa quota and is the largest single source of pilgrims, with more than 200,000 people contributing about 10% of total Hajj pilgrims [2]. The increasing number of pilgrims combined with extreme physical stressors such as sun exposure, heat, thirst, traffic jams, and steep inclines for a long period increases the health risks and exacerbation of underlying conditions [3].

Cardiovascular disease (CVD), including coronary heart disease, hypertension, congestive heart failure, and arrhythmias, is the leading cause of morbidity and mortality during Hajj [4]. It accounts for about 25% of hospitalizations and 64% of ICU admissions among pilgrims in Hajj [5, 6]. Cardiovascular disease was reported as the most common cause of death during Hajj, accounting for 66% of all deaths (446 deaths) of 206,831 Indonesian pilgrims in Hajj 2008 [2]. Due to the high morbidity and mortality of CVD among Hajj pilgrims, it is important to know the risk factors and their significance in increasing the need for hospitalization and mortality. This study aims to identify the impact of classical cardiovascular risk factors on hospitalization requirements among pilgrims from East Java during the 2017, 2018, and 2019 Hajj.

## 2. Methods

### 2.1. Data Collection

**Figure 1:**
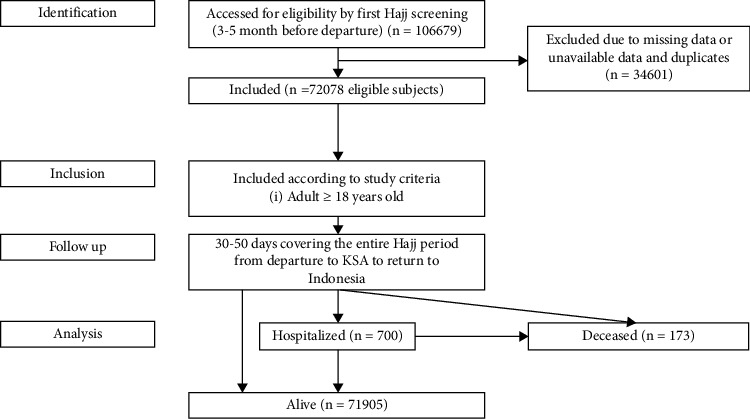
Flowchart of the study design.

The design of this study was a retrospective cohort, noninterventional study. The sample population was all Hajj pilgrims from East Java, Indonesia, from 2017 to 2019. The follow-up period is 30–50 days (median 43 days) covering the entire Hajj period from departure to KSA to return to Indonesia (Figure 1). Data on risk factors were obtained from the Hajj medical service records carried out by trained health workers during the pre-embarkation medical assessment. During predeparture, pilgrims undertake a medical test to receive a meningitis vaccine and confirm travel fitness. Pilgrims aged 60 years or older and had at least one preexisting medical condition (e.g., hypertension and diabetes mellitus) were classified as high risk. Variables extracted from predeparture screening records include demographic data of name, age, gender, home address, and physical examination data, including blood pressure measurement and body mass index (BMI) calculation. Hypertension was defined following the guidelines from the European Society of Cardiology (ESC) 2018 with office systolic blood pressure (SBP) values of 140 and/or diastolic blood pressure (DBP) of 90 mmHg. The classification of hypertension is divided into grade 1 with SBP 140–159 mmHg and/or DBP 90–99 mmHg, grade 2 with SBP 160–179 mmHg and/or DBP 100–109 mmHg, and grade 3 with SBP 180 mmHg and/or DBP 110 mmHg [7]. Obesity was defined based on BMI following the World Health Organization (WHO) classification for overweight and obesity recommendation for Asian population. Participants with BMIs ranging from 18.5 to 22.9 kg/m^2^ were categorized as normal, BMIs less than 18.5 kg/m^2^ were categorized as underweight, BMIs ranging from 23.0 to 24.9 kg/m^2^ were categorized as overweight, BMIs ranging from 25 to 29.9 kg/m^2^ were categorized as obesity class I, and BMIs ≥ 30 kg/m^2^ were categorized as obesity class II [8]. Postprandial blood sugar and the history of cigarette smoking were extracted from the medical report. Diabetes was defined according to the American Diabetes Association (ADA) criteria as a self-reported diagnosis determined previously by a healthcare professional or among participants without self-reported diabetes using an oral glucose tolerance test with a two-hour plasma glucose 200 mg/dL (11.1 mmol/L) [9].

The diagnosis of hospitalization during the Hajj period from 2017 to 2019 was obtained from the medical report. The cause of death was obtained from the hospital or flight doctor death certificate. Hospitalizations and deaths during the Hajj period from 2017 to 2019 were documented by the Indonesian public health team based in Saudi Arabia. Diagnosis of hospitalization and mortality data were coded according to the International Classification of Diseases-10 (ICD-10) coding.

### 2.2. Classical Cardiovascular Risk Factors

Classical cardiovascular risk factors were identified in the Framingham Heart Study as conferring increased risk of CVD in the general population [10]. Classical cardiovascular risk factors analyzed in this study were age, gender, hypertension, diabetes, smoking, and obesity. Blood pressure (BP) determination was made using a periodically calibrated mercury sphygmomanometer. Diabetes was diagnosed at a two-hour blood sugar greater than or equal to 200 mg/dl of the oral glucose tolerance test (OGTT) [11].

### 2.3. Statistical Analysis

All statistical analyses were performed using SPSS 20.0. The normal distribution of data was evaluated using the Kolmogorov–Smirnov test. Continuous variables were presented as median and 25th/75th percentiles, while categorical variables were presented as absolute frequencies and percentages [12]. Pearson's Chi-square or Fisher's exact tests were used to compare univariate continuous variables, and independent *T*-tests were used to compare univariate numeric categorical variables. A *P* value of <0.05 was considered statistically significant. Furthermore, multivariate analysis was performed using logistic regression to identify which classical cardiovascular risk factors predict hospitalization and mortality among Hajj pilgrims. The variables included in the logistic regression analysis were variables with *P* value < 0.25 in bivariate analysis. Receiver operating characteristic (ROC) curves were constructed for classical cardiovascular risk factors and two outcomes of hospitalization and mortality.

### 2.4. Ethics Approval

The clinical and epidemiological data collection was submitted for ethical review to the Ministry of Health of the Republic of Indonesia with the approval number of HJ.01.03/I/2900/2020.

## 3. Results

### 3.1. Characteristics of Patients

Of the 106680 subjects obtained from pilgrims attending the Hajj period from 2017 to 2019, 34602 (32.43%) were excluded from the analysis due to incompleteness in demographic data, physical examination, laboratory results, or patient outcomes. The final sample was therefore composed of 72078 eligible subjects in the study (Table 1) with 21488, 23865, and 26725 pilgrims, respectively, from the 2017, 2018, and 2019 Hajj years. Of these, 33807 (46.9%) were men and 38271 (53.1%) were women. All pilgrims were aged ≥ 18 years with a median age of 54 years (47–61), and the majority (35%) aged between 50 and 59 years. From the three Hajj year periods from 2017 to 2019, there were significant differences in age groups, prevalence of hypertension, BMI distribution, average fasting blood sugar level, and smoking history. In addition, there were also differences in the hospitalization rate and mortality of the Hajj participants from 2017 to 2019. The highest reported hospitalization rate was in the 2019 Hajj year, but the highest mortality rate was in 2017.

A total of 42446 pilgrims (58.9%) were classified as high risk due to underlying health conditions such as hypertension, diabetes, or if they were aged 60 years or older. An increase in BMI was also common in pilgrims, with 13922 (19.3%) subjects being overweight and 36080 (50.1%) subjects being diagnosed as obese. In addition, smoking history was recorded in 10389 (14.4%) subjects, with the majority being men (10202 subjects; 98.2%)

### 3.2. Factors Contributing to the Hospitalization in Pilgrims

During 2017–2019, 700 out of 72078 pilgrims required hospitalization during Hajj in Saudi Arabia (Table 2). The overall hospitalization rate is 971 per 100,000 pilgrims. Men were associated with increased hospitalization than women (1.18% vs. 0.80%, *P* < 0.001). Pilgrims aged 60 years or older (2.17%) also required more hospitalization compared with younger ones, and the rates significantly increased with increasing age (*P* < 0.001). Hospitalization was required for 4.44% of patients with hypertension (versus 0.79% of patients without hypertension; *P* < 0.001) and 2.28% of patients with diabetes (versus 0.82% of patients without diabetes, *P* < 0.001). Obese (BMI ≥ 30) and underweight (BMI < 18.5) pilgrims had the highest percentage of hospitalization (3.18% and 3.17%, respectively) compared with pilgrims with normal BMI (BMI 18.5–24.9; 1.09%; *P* < 0.001). There was no association between smoking history and hospitalization among pilgrims (*P*=0.905).

### 3.3. Factors Contribute to Mortality in Pilgrims

**Figure 2:**
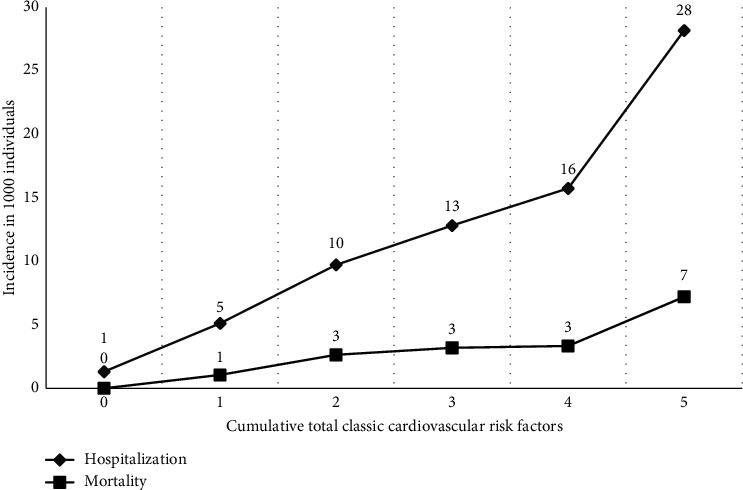
The cumulative total of classical cardiovascular risk factors with the incidence of hospitalization and mortality (in 1000 individuals).

During 2017–2019, 173 out of 72078 pilgrims died during Hajj in Saudi Arabia. The overall death rate is 240 deaths per 100,000 pilgrims (Table 2). The mortality rate was higher in men (144 deaths per 100,000 pilgrims) than in women (96 deaths per 100,000 pilgrims, *P* < 0.001). The mortality rate was highest in those aged 60 years or older (593 per 100,000 pilgrims), and rates significantly increased with increasing age (*P* < 0.001). Mortality was found in 0.29% of patients with hypertension (versus 0.21% of patients without hypertension; *P* < 0.001) and in 0.55% of patients with diabetes (versus 0.20% of patients without diabetes, *P* < 0.001). Underweight pilgrims (BMI < 18.5) had the highest percentage of mortality (0.79%) followed by pilgrims with normal BMI (BMI 18.5–24.9; 0.25%) and overweight and obese pilgrims (BMI ≥ 25; 0.16%; *P* < 0.001). There was no association between smoking history and mortality among pilgrims (*P*=0.839). In addition, when combined, the cumulative total of classical cardiovascular risk factors increases with the incidence of hospitalization and death (Figure 2).

**Figure 3:**
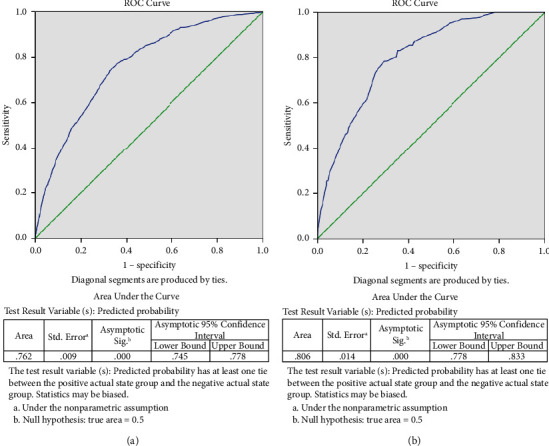
Area under the curve for the association between classical cardiovascular (CV) risk factors and hospitalization (a) and mortality (b)

Multivariate analysis using logistic regression showed that male gender, age > 50 years, hypertension grades II-III, diabetes, overweight, and obesity were associated with a higher risk of hospitalization. Moreover, several variables such as male gender, diabetes, and overweight, but not obesity, were associated with higher risk of mortality (Table 3). The area under the ROC curve (AUC) for male gender, age, hypertension, diabetes, BMI and risk of hospitalization was 0.762. The AUC for male gender, diabetes, BMI, and mortality was 0.806 (Figure 3).

### 3.4. Diagnosis during Hospitalization and Cause of Mortality

**Figure 4:**
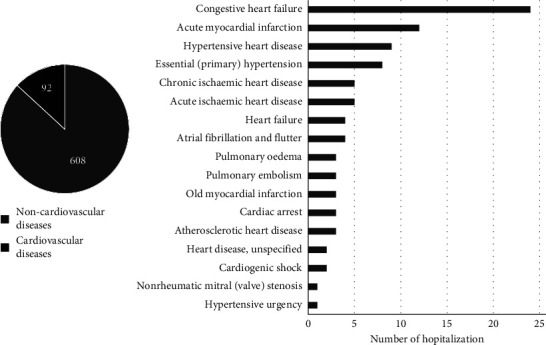
Early diagnosis during hospitalization among Hajj pilgrims.

**Figure 5:**
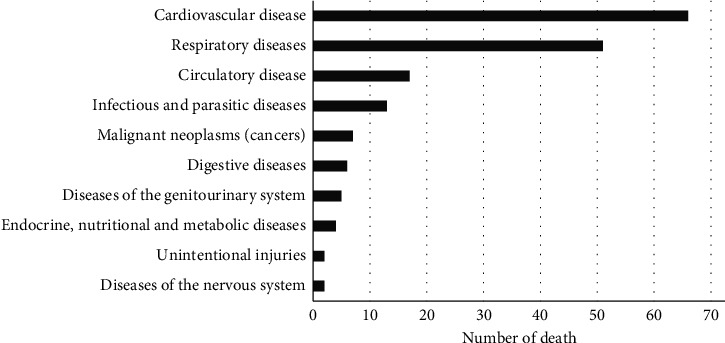
The cause of death according to the hospital/flight doctor death certificate.

Of all hospitalized patients (*n* = 700 pilgrims), 92 patients (13.1%) had an initial diagnosis of CVD (Figure 4). The top three CVDs leading to hospitalization were congestive heart failure (24 pilgrims), acute myocardial infarction (12 pilgrims), and hypertensive heart disease (9 pilgrims). Meanwhile, CVD is the main cause of mortality for pilgrims (38.2%), followed by respiratory diseases (29.5%), circulatory diseases (9.8%), and infectious and parasitic diseases (7.5%) (Figure 5).

## 4. Discussion

Over the last few decades, CVD has emerged as an important cause of hospital admission and death among Hajj pilgrims. Especially pilgrims with preexisting heart disease are at high risk of experiencing physical stress leading to ischemia [4]. A cross-sectional study analyzing admissions to 1487 beds and 104 intensive care units (ICUs) from three hospitals in Arafat and four hospitals in Mena was conducted during the Hajj 2004. This study showed that CVD accounted for nearly 25% of admissions, including congestive heart failure, hypertension, ischemic heart disease, and arrhythmias. Furthermore, it also showed that about 64% of ICU admissions were due to CVD, which was dominated by left ventricular failure and myocardial infarction. In addition, more than 70% of these ICU patients had underlying diseases that required medical attention, with more than half of the comorbidities being CVDs such as congestive heart failure, hypertension, and ischemic heart disease [5, 6]. Another prospective cohort study from four hospitals in Mena during the 2009 Hajj period showed that CVD (23.6%) was the second highest cause of critically ill patients following severe infections as the most prevalent cause of the critical condition (46.5%) with a diagnosis of myocardial infarction, atrial fibrillation (4.7%), and cardiogenic shock (2.7%) [13]. CVD was the most common cause of morbidity (34.1%) in patients necessitating admission to a tertiary care hospital in Makkah during the 2005 Hajj [14]. A previous study in Indonesia showed an association between CVD and increased morbidity and hospital admissions among Hajj pilgrims. Moreover, CVD is the most common cause of death during Hajj, accounting for 66% of all deaths (446 deaths) of 206,831 Indonesian pilgrims in Hajj 2008 [2]. Cardiovascular disease during Hajj was also reported by Saudi Arabian Ministry of Health as the most common cause of death compared to other medical diseases, both communicable and noncommunicable diseases [15].

Due to the high morbidity and mortality of CVD among Hajj pilgrims, it is important to know the risk factors and their significance in increasing the need for hospitalization and mortality. In the second half of the twentieth century, many epidemiological studies identified risk factors for CVD that have contributed to primary prevention. One of the most widely used risk prediction equations is the Framingham model to estimate the 10-year risk of coronary heart disease (CHD). The Framingham model is based on classical risk factors, including age, gender, blood pressure, low-density lipoprotein (LDL) and high-density lipoprotein (HDL) cholesterol levels, smoking, and diabetes [10]. Although, the long-standing, recent evidence based on population risk calculations in the United States using the Framingham 10-year risk estimate for CHD suggests that 75%–85% of CHD can be prevented by avoiding classic risk factors [16].

Identifying cardiovascular risk factors in Hajj participants is important because it is estimated that the obligation to carry out the rites during Hajj increases the burden on cardiac function, especially in pilgrims with underlying CVD. Sudden strenuous physical activity results in stressful exercises and triggers several mechanisms, including the decreased venous return and decreased cardiac output that may lead to an acute cardiovascular attack. This process is counterproductive and can compromise disease management and, in extreme cases, can be fatal [4]. This condition is exacerbated by the heat in which sweating causes fluid loss, leading to dehydration and hypovolemia, decreased cardiac output, and loss of body fluids into the interstitial fluid spaces with subsequent cardiovascular collapse. If heat stress continues beyond this compensatory stage, the central venous pressure falls sharply, causing a further rise in core body temperature with subsequent failure of thermoregulatory mechanisms and leading to heatstroke [17].

In multivariate analysis, diabetes consistently and significantly increased the risk of hospitalization and death of Hajj participants in this study. A systematic review showed that the prevalence of diabetes is one of the most common comorbidities found in Hajj pilgrims with a prevalence of 5% [18]. Diabetes and related complications have also been reported as a very influential risk factor and are one of the main causes of hospitalization and mortality among pilgrims [19]. In addition, the male gender is also more often found in hospitalized and deceased pilgrims. This was also found in a report from Indian Hajj Pilgrims where of the 163 deaths, 68.7% were male and the most common terminal event was cardiorespiratory arrest [20]. Interestingly, this research also showed that underweight pilgrims (BMI < 18.5) had a higher rate of hospitalization (3.18%) and mortality (1.10%) compared with normal BMI, although this risk factor failed to show a significant impact on multivariate analysis. This unexpected result of the increased mortality risk in the underweight group can be associated with various clinical factors, such as poor nutritional status, sarcopenia, and aging in underweight population. Previous research also suggested that underweight may be a risk factor for CVD disease with 19.7% greater risk than the normal weight [21].

A study in Iran provided intervention to prospective pilgrims with a history of CVD. These interventions include cancellation of prospective Hajj pilgrims with severe CVD (history of recent myocardial infarction, unstable angina, advanced heart failure, and uncontrolled hypertension) and provision of adequate therapy and monitoring for other patients with stable angina, mild heart failure, controlled hypertension, and other cardiovascular disorders. It showed that mortality and hospitalization rates were significantly reduced in the intervention population. The Iranian study and the results of this study support the need for health screening before departure, especially for classical cardiovascular risk factors. Pilgrims with severe CVD should be excluded from Hajj. Other pilgrims with CVD or those at risk of CVD need appropriate intervention and monitoring during Hajj to reduce hospitalization and mortality rates in Hajj pilgrims.

When compared to studies with similar areas and periods to this study, the results were similar. A study that screened cardiovascular risk factors in adults aged 40 years and in Malang, East Java, in 2016-2017, showed that out of 22,093 participants, 6,455 (29.2%) had high cardiovascular risk with an estimated 10-year CVD risk of ≥30%. In this study, high cardiovascular risk was defined as the presence of coronary heart disease, stroke, or other atherosclerotic disease [22]. Another study analyzing the report of Riskesdas 2013, a nationally representative survey conducted by the Indonesian Ministry of Health in 2013, estimated the coronary heart disease (CHD) burden caused by five major and modifiable vascular risk factors: smoking, hypertension, diabetes, increased total cholesterol, and overweight. The results of this study showed hypertension as a major vascular risk factor (20%–25% of all CHD) and smoking in men explains most of the vascular events (25% of CHD) [23].

### 4.1. Study Limitation

Although this study uses large data of pilgrims from three time periods of Hajj (2017–2019), the sample size only represents a portion of the overall Hajj pilgrims in Indonesia. In addition, the retrospective cohort design using only univariate analysis limits the generalizability of the findings. Data on risk factors were collected from predeparture screening reports so that the responses obtained were susceptible to information bias. The multivariate analysis showed that overweight, but not obesity, significantly contributed to mortality. This may be due to the low mortality rate of 173 from the total 71904 (0.24%) Hajj participants; thus, the distribution may not be representative.

## 5. Conclusion

Pilgrims with classical cardiovascular risk factors were associated with increased hospitalization and mortality. Cardiovascular disease accounted for about 13% of hospitalization and the majority (38.2%) of the causes of death. These findings indicate the need to identify classical cardiovascular risk factors before departure. Cardiac prevention approaches and monitoring are needed for pilgrims with CVD or those at risk of CVD during the Hajj period to reduce hospitalization and death associated with cardiovascular events.

## Figures and Tables

**Table 1:** Characteristics of the study population of Hajj pilgrims from East Java, Indonesia, from 2017 to 2019.

Variable	2017 (*n* = 21488)	2018 (*n* = 23865)	2019 (*n* = 26725)	*P* value (*χ*^2^ test/ANOVA)	Post hoc test	*P* value (Mann–Whitney)
Men	10018 (46.6)	11254 (47.2)	12535 (46.9)	0.521		
Women	11470 (53.4)	12611 (52.8)	14190 (53.1)			
Age (years)	54.0 (11.3)	53.8 (10.8)	54.14 (11.8)	0.145		
18–40	2369 (11.0)	2473 (10.4)	3086 (11.5)	<0.001		
41–49	5082 (23.7)	5499 (23.0)	5902 (22.1)			
50–59	7490 (34.9)	8860 (37.1)	9442 (35.3)			
≥60	6547 (30.5)	7033 (29.5)	8295 (31.0)			
SBP (mmHg)	127.3 (20.4)	127.9 (20.5)	128.4 (21.0)	<0.001	2017 vs. 2018	0.001
					2017 vs. 2019	<0.001
					2018 vs. 2019	0.045
DBP (mmHg)	80.4 (10.2)	80.5 (10.2)	80.3 (10.4)	0.006	2017 vs. 2018	0.232
					2017 vs. 2019	0.660
					2018 vs. 2019	0.002
Nonhypertension	10000 (46.5)	15376 (64.4)	1282 (4.8)	<0.001		
HT grade I	7612 (35.4)	5491 (23.0)	6936 (26.0)			
HT grade II	2785 (13.0)	2200 (9.2)	5129 (19.2)			
HT grade III	1091 (5.1)	798 (3.3)	3614 (13.5)			
BMI	25.3 (4.6)	25.3 (4.5)	25.3 (4.6)	0.349		
Underweight	974 (4.5)	953 (4.0)	1282 (4.8)	0.002		
Normal	5686 (26.5)	6245 (26.2)	6936 (26)			
Overweight	4130 (19.2)	4663 (19.5)	5129 (19.2)			
Obesity class I	7885 (36.7)	8872 (13.1)	9764 (36.5)			
Obesity class II	2813 (13.1)	3132 (13.1)	3614 (13.5)			
PPG levels (mg/dL)	136.9 (63.4)	138.0 (62.4)	138.6 (63.8)	<0.001	2017 vs. 2018	<0.001
					2017 vs. 2019	<0.001
					2018 vs. 2019	0.776
Diabetes	2340 (10.9)	2556 (10.7)	2985 (11.2)	0.247		
Smoking history	2987 (13.9)	3420 (14.3)	3982 (14.9)	0.007		
Hospitalization	173 (0.8)	230 (1.0)	297 (1.1)	0.003		
Mortality	77 (0.4)	42 (0.2)	54 (0.2)	<0.001		

**Table 2:** Univariate analysis for hospitalization and mortality of Hajj pilgrims from East Java, Indonesia, from 2017 to 2019.

Variable	Hospitalization		*P* value	Mortality		*P* value
	No, *n* = 71378	Yes, *n* = 700		Alive, *n* = 71904	Death, *n* = 173	
Men	33412	395	<0.001	33703	104	<0.001
Women	37966	305		38202	69	
Age (years)	53.9 (11.3)	64.2 (11.1)	<0.001	54.0 (11.3)	66.8 (10.2)	<0.001
Age group						
18–40	7914	14	<0.001	7928	0	<0.001
41–49	16436	47		16474	9	
50–59	25618	174		25757	35	
≥60	21410	465		21746	129	
SBP (mmHg)	127.8 (20.6)	136.1 (13)	<0.001	127.9 (20.7)	134.3 (22.4)	<0.001
DBP (mmHg)	80.4 (10.3)	82.9 (11.1)	<0.001	80.4 (10.3)	82.5 (11.1)	0.005
Non-HTN	42351	333	<0.001	42597	87	0.001
HTN grade I	18897	198		19050	45	
HTN grade II	7282	119		7375	26	
HTN grade III	2848	50		2883	15	
BMI	25.3 (4.6)	23.7 (5.3)	<0.001	25.3 (4.6)	22.9 (5.5)	<0.001
Underweight	3110	99	<0.001	3174	35	<0.001
Normal	18634	233		18810	57	
Overweight	13803	119		13898	24	
Obesity class I	26355	166		26482	39	
Obesity class II	9476	83		9541	18	
PPG levels (mg/dL)	137.6 (62.8)	169.3 (90.6)	<0.001	137.8 (63.1)	169.4 (97.6)	<0.001
Diabetes						
Yes	7705	176	<0.001	7838	43	<0.001
No	63672	524		64067	130	
Smoking history						
Yes	10287	102	0.905	10365	24	0.839
No	61091	598		61540	149	

**Table 3:** Multivariate analysis using logistic regression for risk factors of hospitalization and mortality Hajj pilgrims from East Java, Indonesia, from 2017 to 2019.

Variable	Hospitalization		
	Odds ratio	95% CI	*P* value
Men	1.334	1.146–1.554	<0.001
Age group (years)			
18–40	Reference		
41–49	1.546	0.850–2.812	0.153
50–59	3.262	1.856–5.640	<0.001
≥60	8.906	5.204-15.244	<0.001
Hypertension			
Non-HTN	References		
HTN grade I	1.055	0.882–1.263	0.559
HTN grade II	1.546	1.247–1.917	<0.001
HTN grade III	1.657	1.222–2.247	<0.001
Diabetes	2.552	2.099–3.102	<0.001
BMI			
Normal	Reference		
Underweight	1.031	0.796–1.336	0.816
Overweight	2.244	1.653–3.047	<0.001
Obesity class I	0.788	0.592–1.048	0.102
Obesity class II	0.615	0.471–0.803	<0.001
Men	1.480	1.088–2.014	0.012
Diabetes	2.567	1.808–3.643	<0.001
BMI			
Normal	Reference		
Underweight	1.009	0.588–1.731	0.975
Overweight	2.985	1.660–5.369	<0.001
Obesity class I	0.664	0.358–1.231	0.193
Obesity class II	0.631	0.360–1.106	0.108

## Data Availability

The data used to support the findings of this study are available from the corresponding author upon request.
